# Comparison of the Radiomics Features of Normal-Appearing White Matter in Persons with High or Low Perivascular Space Scores

**DOI:** 10.3390/jimaging12010035

**Published:** 2026-01-08

**Authors:** Onural Ozturk, Sibel Balci, Seda Ozturk

**Affiliations:** 1 Department of Radiology, Northwestern University Feinberg School of Medicine, Chicago, IL 60611, USA; 2 Department of Biostatistics, School of Medicine, Kocaeli University, Izmit 41000, Turkey; 3 Independent Researcher, Indian Head Park, IL 60525, USA

**Keywords:** radiomics, perivascular space, normal-appearing white matter, LASSO, texture analysis

## Abstract

The clinical significance of perivascular spaces (PVS) remains controversial. Radiomics refers to the extraction of quantitative features from medical images using pixel-based computational approaches. This study aimed to compare the radiomics features of normal-appearing white matter (NAWM) in patients with low and high PVS scores to reveal microstructural differences that are not visible macroscopically. Adult patients who underwent cranial MRI over a one-month period were retrospectively screened and divided into two groups according to their global PVS score. Radiomics feature extraction from NAWM was performed at the level of the centrum semiovale on FLAIR and ADC images. Radiomics features were selected using Least Absolute Shrinkage and Selection Operator (LASSO) regression during the initial model development phase, and predefined radiomics scores were evaluated for both sequences. A total of 160 patients were included in the study. Radiomics scores derived from normal-appearing white matter demonstrated good discriminative performance for differentiating high vs. low perivascular space (PVS) burden (AUC = 0.853 for FLAIR and AUC = 0.753 for ADC). In age- and scanner-adjusted multivariable models, radiomics scores remained independently associated with high PVS burden. These findings suggest that radiomics analysis of NAWM can capture subtle white matter alterations associated with PVS burden and may serve as a non-invasive biomarker for early detection of microvascular and inflammatory changes.

## 1. Introduction

The increasing use of Magnetic Resonance Imaging (MRI) for cranial imaging makes detection of intracranial pathologies more frequent. Greater spatial resolution in the brain means MRI is superior to computed tomography for detection of intracranial pathologies. However, this increase in the detection rate of incidental findings without clinical importance or of unknown importance tends to result in increased use of additional radiologic examinations, accompanied by unnecessary follow-ups and an increased workload for radiologists.

The perivascular space (PVS) is located between the adventitial layer of small arterioles or venules and the basal membrane, consisting of glial cells [1]. The physiological role of the PVS is to drain brain-interstitial fluid to the subarachnoid space via perivascular pathways [2]. Dilated PVSs were long thought to be of no clinical importance. Recently, some studies have shown that the size and number of dilated PVSs might be related to intracranial vascular processes, such as cerebrovascular events and small vessel disease [1,3,4,5,6,7]. In patients with vascular dementia, the presence of PVS is increased compared to Alzheimer’s disease. Additionally, increased PVS volume in healthy subjects with white matter hyperintensities might be further evidence to support this hypothesis [3]. In spite of the research into the significance of PVS changes, the relationship between PVS and vascular pathologies remains unclear.

“Radiomics” is the study of a series of quantitative data that can be obtained by assessing digital images, including medical digital images, using pixel-based approaches, such as texture analysis [8]. Evaluation of radiological images is dependent on the attention, knowledge, and experience of the radiologists performing the analysis. Furthermore, the assessment process of a series of radiological images can lead to variability in the evaluation as the process proceeds through the series. Moreover, pixel-based evaluation of different tissues and lesions is not possible for the human eye, regardless of the experience level of the observer. With Radiomics, a large amount of information can be obtained, including pattern recognition (gray and white matter patterns), the relationship between pixels, and spectral analysis of lesions or tissues. Automatic pattern recognition and image analysis software offers the promise of reducing inter-individual and intra-individual assessment variability [9]. Furthermore, pixel-based analysis is promising in terms of identifying image features that cannot be determined by conventional and functional imaging methods in both normal and pathological tissues.

The aim of this study was to compare the radiomics values of normal-appearing white matter (NAWM) in patients with different PVS scores to investigate differences that would not be distinguishable to the naked eye, but which might be related to microvascular processes or hemodynamic changes.

## 2. Materials and Methods

### 2.1. Ethical Committee Approval

The Ethical committee approval was obtained from our institutional board. The study data were obtained retrospectively.

### 2.2. Patient Recruitment

Patients who underwent cranial MRI over a one-month period were screened. The inclusion criteria were being older than 18 years and having adequate MR imaging during the specified study period. Exclusion criteria were as follows: patients with inadequate imaging series (motion artifacts, inadequate image quality, etc.); patients with structural brain injury (patients that have conditions that may lead to significant white matter loss, such as congenital anomalies, large masses with or without peripheral edema [>2 cm in size], postoperative changes, or extensive infarct areas); and patients with widespread white matter involvement who did not have a normal white matter area available to measure at the centrum semiovale level (chemotherapy or radiotherapy-related changes, widespread small vessel disease involvement, widespread demyelinating disease involvement, or metabolic diseases affecting white matter).

### 2.3. MRI Acquisition

The patients were placed in a supine position. An eight-channel Head coil was used, and they were scanned according to routine protocols in a 1.5 Tesla MRI (Gyroscan Intera; Philips Medical Systems, Eindhoven, The Netherlands) and 3 Tesla MRI (Achieva Intera: Philips Medical Systems, Eindhoven, The Netherlands). Both apparent diffusion coefficient (ADC) and fluid attenuation inversion recovery (FLAIR) images were assessed in all study subjects. Routine cranial imaging protocol parameters are shown in Table 1 and Table 2.

#### 2.3.1. Perivascular Space Visual Assessment Score

The final cohort was divided into two equal-sized groups based on the global PVS score; one group with a PVS ≤ 15 was designated Group 1, and the remaining patients with a global PVS score ≥ 16 were designated as Group 2.

Lesions with an oval or round shape, T1 hypointense, T2 hyperintense, showing signal loss in FLAIR images, showing consistent distribution with periarterial anatomy, and with no hyperintense surrounding rim (suggestive of gliosis) were defined as PVS, as previously described [10].

**Figure 1:**
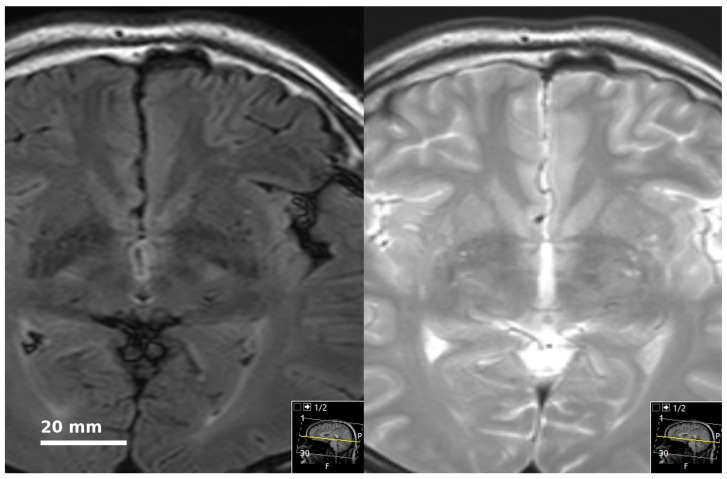
A subject with a PVS score of “2” at the left basal ganglia and “1” at the right basal ganglia.

The PVS score was based on the visual assessment scores indicated in previous studies [1,6,11,12]. PVS scores were calculated separately on each brain side in seven different regions. These regions were: (1) mesencephalon (including upper pons); (2) cerebellum; (3) hippocampus; (4) subinsular region (capsula externa, claustrum, capsula extrema); (5) basal ganglia; (6) frontal lobe; and (7) parieto-occipital lobes. Each region was assessed for the quantity and size of PVS. Thus, in a defined region, 0 PVS scored as 0 points, 1–5 PVS scored as 1 point, 6–10 PVS scored as 2 points, 11 and above PVS scored as 3 points (See Figure 1). In size assessment, the longest diameter of the most prominent PVS determined the size score: PVS <2 mm scored as 1 point; PVS ≥2 mm and <4 mm scored as 2 points; and ≥4 scored as 3 points. Global PVS scores ranging from 0 to 84 points were obtained.

#### 2.3.2. ROI Selection and Feature Extraction

**Figure 2:**
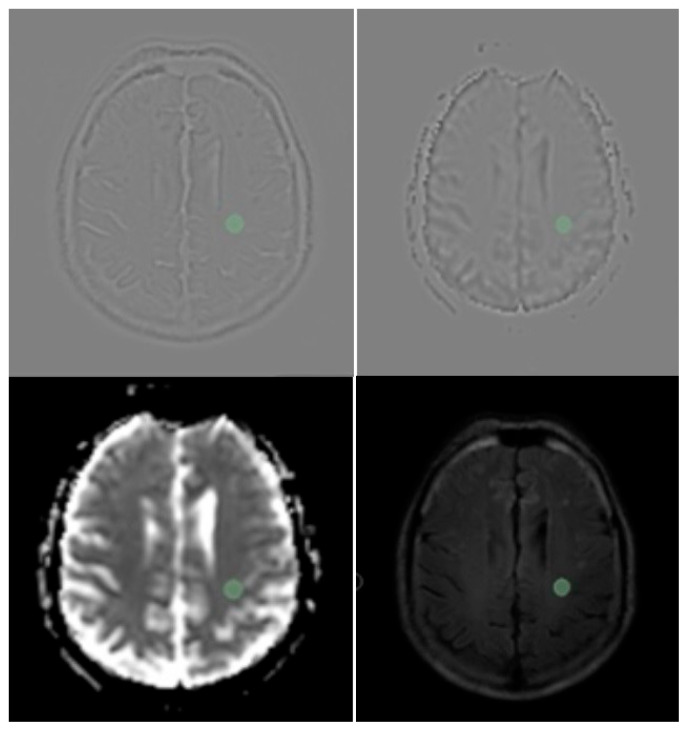
Centrum semiovale sampling of white matter with 3Dslicer software in LoG filtered and unfiltered FLAlR and ADC images. (Note that in FLAlR images sampling Rol (green circle) is at least 1 cm far from T2 hyperintense lesions).

In this study, centrum semiovale radiomics features in patients with different global PVS scores are compared. Normal-appearing centrum semiovale was defined as areas without any signal alterations on T1, T2 and DW images and involving no signal alterations in a 1 cm distance around the specified region of interest (ROI). A circular area with a diameter of 1 cm and meeting the conditions to define “normal appearing centrum semiovale area” was selected for further assessment [11] (Figure 2).

Data of the patients was exported into 3DSlicer version 4.9.0 [13] software in DICOM file format. This allowed 116 features to be extracted using the published “Radiomics” extension. Detailed descriptions of these features were published by Zwanenburg et al. [14].

#### 2.3.3. Feature Selection and Radiomics Signature Definition

Radiomics features for both FLAIR and ADC sequences were selected using Least Absolute Shrinkage and Selection Operator (LASSO) regression during the initial model development phase. To avoid outcome-driven feature re-selection and optimistic bias, LASSO was not repeated in the revised analysis. Instead, the predefined radiomics signatures were treated as fixed predictors.

For the FLAIR sequence, the radiomics signature consisted of seven texture features: DependenceEntropy, LargeDependenceLowGrayLevelEmphasis, ClusterShade, Contrast, LowGrayLevelRunEmphasis, GrayLevelNonUniformity_B, and LowGrayLevelZoneEmphasis.

For the ADC sequence, the radiomics signature consisted of five features: DependenceEntropy, LargeDependenceLowGrayLevelEmphasis, ClusterShade, LowGrayLevelRunEmphasis, and GrayLevelNonUniformity_B.

Radiomics scores were constructed by standardizing each feature using z-score normalization and combining them into a composite radiomics score for each sequence.

#### 2.3.4. Statistical Analysis

Statistical analyses were performed using SPSS (version 20.0; IBM Corp., Armonk, NY, USA). Normality was assessed using the Shapiro–Wilk and Kolmogorov–Smirnov tests.

Patients were dichotomized into low (≤15) and high (≥16) PVS burden groups. The primary objective was to evaluate whether radiomics signatures were associated with high PVS burden independent of age and MRI field strength.

Multivariable binary logistic regression models were constructed separately for FLAIR and ADC radiomics scores, with the PVS group as the dependent variable and radiomics score as the predictor of interest, adjusting for age and scanner field strength (1.5 T vs. 3 T). Results are reported as adjusted odds ratios (ORs) with 95% confidence intervals (CIs).

Discriminative performance was evaluated using receiver operating characteristic (ROC) analysis with area under the curve (AUC). Scanner-specific ROC analyses were performed separately for 1.5 T and 3 T subgroups. Sensitivity analyses were conducted by repeating the adjusted models within a single field-strength cohort. A two-sided *p*-value < 0.05 was considered statistically significant.

## 3. Results

There were 251 cranial examinations in the specified period. Of these, 31 had a structural brain injury, 30 had an inadequate MRI series, and 30 had evidence of widespread white matter involvement and were therefore excluded. Therefore, the final study cohort consisted of 160 evaluated patients.

Mean global PVS scores were 8.64 ± 3.54 and 26.03 ± 8.74 for Group 1 and Group 2, respectively. Mean ± SD ages for the groups were as follows: Group 1 = 43.80 ± 15.18 years and Group 2 = 53.76 ± 13.72 years, which were significantly different. Fifty-four of the patients were scanned at 1.5 T and 106 at 3 T. Increasing age was positively correlated with global PVS score (*p* < 0.05).

In multivariable logistic regression adjusting for age and MRI field strength, the FLAIR radiomics score remained independently associated with high PVS burden (adjusted OR = 2.63, 95% CI 1.78–4.01, *p* < 0.001).

Similarly, the ADC radiomics score was independently associated with high PVS burden after adjustment (adjusted OR = 2.41, 95% CI 1.64–3.69, *p* < 0.001).

The region with the most PVS was the frontal region, with an average PVS per patient of 5.26. In descending order after the frontal region, the regions with average values of PVS per patient were the basal ganglia (4.78); the parieto-occipital lobes (2.36); the subinsular region (1.98); the hippocampi (1.42); the cerebellum (0.98); and the mesencephalon, including upper pons (0.36).

**Figure 3:**
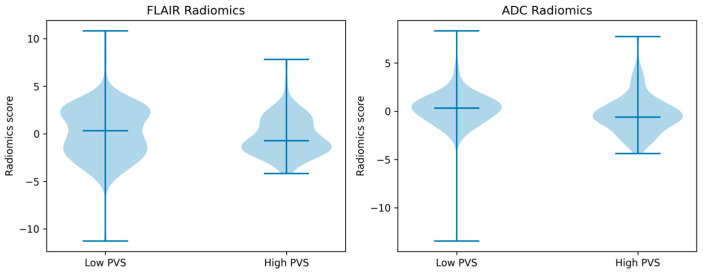
Distribution of radiomics scores derived from normal-appearing white matter in patients with low and high perivascular space (PVS) burden. Violin plots illustrate the distribution of radiomics scores for FLAIR-based and ADC-based radiomics models, with central markers indicating median values.

Radiomics signatures derived from predefined LASSO-selected features were evaluated for their ability to differentiate high and low PVS burden. Radiomics score distributions for patients with low and high PVS burden are shown in Figure 3, demonstrating separation between groups.

**Figure 4:**
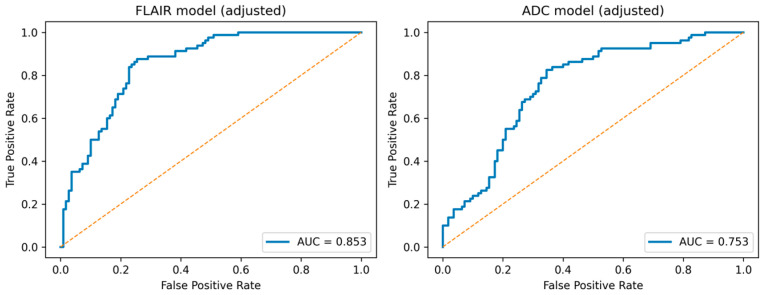
Receiver operating characteristic (ROC) curves demonstrating the discriminative performance of radiomics scores derived from FLAIR and ADC images for differentiating high vs. low perivascular space (PVS) burden.

The FLAIR radiomics model achieved an AUC of 0.853, while the ADC radiomics model achieved an AUC of 0.753 (Figure 4). Scanner-stratified ROC analyses demonstrated comparable discriminative performance across field strengths.

**Figure 5:**
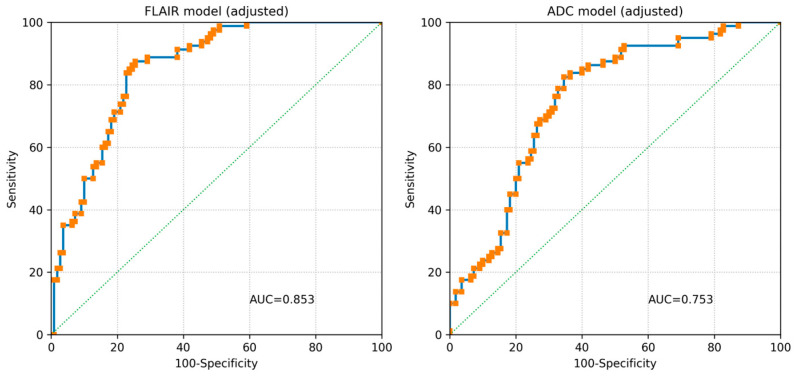
Sensitivity vs. 100–specificity plots illustrating diagnostic performance of radiomics scores across thresholds for FLAIR-based and ADC-based radiomics models.

Results remained directionally consistent in sensitivity analyses restricted to a single field strength, supporting the robustness of the findings (Figure 5).

## 4. Discussion

Since high imaging quality is obtained with MRI when assessing cranial diseases, and these images are suitable for detailed analysis of the PVSs, there have been some attempts to relate PVS to cranial vascular processes; however, the clinical significance of increased PVS remains to be clarified [1,4]. Our study suggests a potentially increased risk of vascular processes with a higher number and size of PVS, which makes the detection and evaluation of that feature more important. Radiomics is a relatively new method using pixel-based changes in digital diagnostic images that goes beyond traditional radiologic assessments to identify possible changes in “normal” appearing tissues.

In this study, we attempted to use radiomics to identify possible micro-changes in NAWM in patients with high PVS scores compared to patients with lower PVS scores. Because the PVS burden increased with age, age was included as a covariate in all multivariable models. Importantly, radiomics signatures derived from NAWM remained associated with PVS burden after age and scanner adjustment, suggesting that these features capture microstructural alterations not fully explained by aging alone. MRI field strength is known to influence radiomics feature values. To address this, scanner-adjusted models, scanner-stratified ROC analyses, and sensitivity analyses were performed, demonstrating that the observed associations were robust across field strengths.

Shao et al. [7] investigated white matter in patients over 60 years of age who underwent cranial MRI and were followed up for at least one year. Using radiomics techniques, these authors were able to identify differences between patients who developed or did not develop white matter hyperintensities during follow-up. They suggested that radiomics may enable the earlier detection of white matter hyperintensities, which is consistent with our findings.

Loizou et al. [15] compared radiomics features of multiple sclerosis (MS) lesions with NAWM radiomics features of patients with clinically isolated syndrome and healthy controls. There were multiple features showing significant alterations between the two groups. These results suggest that radiomics analysis may be capable of identifying changes in NAWM, undetectable with traditional image analysis methods.

Kovalev et al. [16] assessed NAWM radiomics features of young males and females. Statistically significant changes were found between males and females at the same ages. The white matter structure of young women was found to be more regular compared to that of young males. Furthermore, as age increased, NAWM features showed significant changes compared to those of younger adults. For both genders, white matter structure became more irregular with age. Detection of subtle changes in NAWM by using radiomics supports our findings in this study.

Tozer et al. [17] compared “texture” analysis features of NAWM, normal-appearing gray matter and characteristic lesions in patients with MS, patients with clinically isolated syndrome and in a healthy control group. Feature comparison between controls and the clinically isolated syndrome patients showed no significant differences. However, there were multiple significant differences between features found in images from MS patients and the other two groups. These findings might prove that there are ongoing inflammatory changes in these patients and support our findings that radiomics may be used as a tool to detect these changes.

One of the strengths of our study was that images were obtained using routine protocols, and only a single ROI was used for the assessment of NAWM, which leads to a rapid evaluation. These study settings were chosen to assess if a “radiomics” process could be easily performed in daily practice with simple applications added to workstations without any postprocessing on images. However, our findings need to be confirmed in further studies using a similar study design.

The major limitation of our study is that different MRI acquisition values were used due to the magnetic power of MRI devices. This might affect the detection level of PVS as well as the radiomics data. Another limitation was that the study lacked intra- and inter-observer correlation data for manual PVS scoring.

We preferred to assess radiomics parameters without performing any postprocessing, aiming for the usage of this data in daily routine. A major strength of this study is the use of predefined radiomics signatures derived using LASSO regression, followed by independent adjusted evaluation, which minimizes feature-selection bias.

In further similar studies, if different image protocols are used, it may be beneficial to use image normalization processes to minimize variation and obtain more accurate results. Limitations include the use of a single ROI, lack of inter-observer PVS scoring reproducibility, and absence of detailed vascular risk factor data due to the retrospective design. Image normalization could also enable comparison of data sets with and without postprocessing. Finally, we suggest that for further similar studies, long-term follow-up and evaluation of patients showing feature alterations in NAWM related to higher PVS score may provide evidence of the clinical utility of the assessment of PVS in all patients who undergo cranial MRI.

## 5. Conclusions

The detected differences in radiomics features in the NAWM in patients with low and high PVS scores may reveal differences in PVS volume and number, as well as white matter hyperintensities undetectable at the macroscopic level. The easy application of the technique increases its potential usage in daily routine.

These findings might propose that microstructural changes are present even in the normal-appearing MRI settings. Along with previous studies, radiomics might be effective in depicting structural changes due to microvascular processes and inflammatory processes. Further, similar studies are needed to expand on these initial findings for the potential use of radiomics features as biomarkers.

## Figures and Tables

**Table 1:** MRI acquisition parameters for 1.5 T field power.

Sequence	FLAIR	ADC
TR	11,000 ms	4000 ms
TE	140 ms	80 ms
TI	2800 ms	-
Slice Thickness	5 mm	5 mm
Slice Gap	0.5 mm	0.5 mm

**Table 2:** MRI acquisition parameters for 3 T field power.

Sequence	FLAIR	ADC
TR	11,000 ms	4000 ms
TE	140 ms	80 ms
TI	2500 ms	-
Slice Thickness	5 mm	5 mm
Slice Gap	0.5 mm	0.5 mm

## Data Availability

The data presented in this study are available from the corresponding author upon reasonable request.

## Abbreviations

ADC	Apparent diffusion coefficient
AUC	area under curve
DWI	diffusion weighted imaging
FLAIR	fluid attenuation inversion recovery
LASSO	Least absolute shrinkage and selection operator
MRI	Magnetic resonance imaging
NAWM	normal-appearing white matter
PVS	perivascular space
ROC	Receiver operating characteristics
